# Health screening, cardiometabolic disease and adverse health outcomes in individuals with severe mental illness

**DOI:** 10.1192/bjo.2019.76

**Published:** 2019-11-08

**Authors:** Robert Pearsall, Richard J. Shaw, Gary McLean, Moira Connolly, Kate A. Hughes, James G. Boyle, John Park, Daniel J. Smith, Daniel Mackay

**Affiliations:** Consultant Psychiatrist and Honorary Clinical Senior Lecturer in Psychiatry, Institute of Health and Wellbeing, University of Glasgow, UK; Research Associate, Institute of Health and Wellbeing, University of Glasgow, UK; Consultant Psychiatrist, Department of Psychiatry, Gartnavel Royal Hospital, Glasgow, UK; Consultant Endocrinologist, Physician and Honorary Senior Lecturer, Department of Medicine, Glasgow Royal Infirmary, UK; Consultant Diabetologist and Honorary Clinical Associate Professor, School of Medicine, University of Glasgow, UK; RN (Mental Health), Lead Research Nurse, Department of Psychiatry, Stobhill Hospital, UK; Professor of Psychiatry, Institute of Health and Wellbeing, University of Glasgow, UK; Reader in Public Health, Institute of Health and Wellbeing, University of Glasgow, UK

**Keywords:** Screening, diabetes, antipsychotic medication, schizophrenia, physical health

## Abstract

**Background:**

Poor physical health in severe mental illness (SMI) remains a major issue for clinical practice.

**Aims:**

To use electronic health records of routinely collected clinical data to determine levels of screening for cardiometabolic disease and adverse health outcomes in a large sample (*n* = 7718) of patients with SMI, predominantly schizophrenia and bipolar disorder.

**Method:**

We linked data from the Glasgow Psychosis Clinical Information System (PsyCIS) to morbidity records, routine blood results and prescribing data.

**Results:**

There was no record of routine blood monitoring during the preceding 2 years for 16.9% of the cohort. However, monitoring was poorer for male patients, younger patients aged 16–44, those with schizophrenia, and for tests of cholesterol, triglyceride and glycosylated haemoglobin. We estimated that 8.0% of participants had diabetes and that lipids levels, and use of lipid-lowering medication, was generally high.

**Conclusions:**

Electronic record linkage identified poor health screening and adverse health outcomes in this vulnerable patient group. This approach can inform the design of future interventions and health policy.

## Background

The physical health of people with severe mental illness (SMI) continues to be a major public health concern.^1^ The standardised mortality rate for individuals with schizophrenia and bipolar disorder is twice that of the general population.^2^ The life expectancy of individuals with SMI is 18.7 years shorter for men and 16.3 years for women,^3^ with cardiovascular disease the leading cause of death.^4^ Despite a consistent fall in premature mortality within the general population during recent decades^5^ the widening differential gap in mortality suggests that people with schizophrenia and bipolar disorder have not shared in the improvements in health seen in the population at large.^2^

It is well documented that individuals with schizophrenia have high levels of cardiovascular disease,^6^ metabolic disease,^7^ diabetes^7^ and respiratory illness.^8^ Explanations for the high levels of morbidity are complex and multifactorial including a genetic predisposition, medication effects, environmental factors with high levels of smoking,^6^ obesity^9^ and sedentariness^10^ all contributing to poor physical health within this population.

Screening for disorders such as diabetes, cardiovascular disease and the risk factors associated with these conditions is known to be suboptimal within this population. Barnes *et al*^11^ conducted an audit of screening for metabolic syndrome in UK mental health services in 2006. They found that within the previous year a blood glucose (or haemoglobin A_1c_ (HbA_1c_)) had been checked in only 28.0% of individuals, plasma lipids in 22.0% and in 6.0% a documented diagnosis of diabetes. In the National Audit of Schizophrenia, Crawford *et al*^12^ found that at the time point of assessment (2011) 59.8% of patients had a plasma glucose recorded, 54.1% had a plasma cholesterol level recorded and 64.6% a blood pressure recording within the last 12 months.

## Existing guidelines on screening individuals with SMI

The National Institute for Health and Care Excellence^13^ have recommended that for people with schizophrenia a fasting blood glucose, HbA_1c_ and blood lipid levels should be measured at intervals of 12 weeks, 1 year and then annually thereafter. For individuals with bipolar disorders, healthcare services are advised to routinely monitor weight and other cardiovascular and metabolic indicators of morbidity, although the frequency of monitoring is not specified.^14^ The Royal College of Psychiatrists^15^ recommend that for people prescribed second-generation antipsychotic medication, body mass index and/or waist circumference, blood pressure, current and previous smoking status, lipids including low-density lipoprotein and high-density lipoprotein (HDL) and random or fasting glucose should all be measured.

## Study aims

The continued development of large electronic record data-sets and data science has offered both feasible and relatively inexpensive approaches to help study real-world data and real-world evidence.^16^ The proliferation of electronic health records offers a number of advantages over standard clinical trial data such as helping to quantify levels of diseases such as diabetes in clinical groups that are at high risk and difficult to access within current service structures. Additionally, the availability of new methods of screening for disorders such as using glycosylated haemoglobin in diabetes,^17^ may offer an opportunity to identify and treat populations that may otherwise be missed with existing approaches. Disorders such as diabetes are more common in people with SMI, and early detection may help to develop more effective ways of improving outcomes for this condition.^18^ The aim of our study was to use a novel health informatics and data linkage approach to investigate levels of health screening, cardiometabolic disease and adverse health outcomes in people with SMI over a 2-year period.

## Method

### Data sources

We linked patient records in a retrospective cross-sectional analysis from the Glasgow Psychosis Clinical Information System (PsyCIS)^19^ to the following Scottish Morbidity Records data-sets:^20^ general/acute in-patient and day case, mental health in-patient and day case, NHS Greater Glasgow and Clyde generated blood test results from Scottish Care Information, the Prescriptions Information System, and National Records of Scotland death certification records over a 2-year period to the 12 November, 2015. The PsyCIS data-set uniquely contains clinical and sociodemographic information gathered on over 10 000 patients (patients in active treatment with NHS Greater Glasgow and Clyde adult mental health services on 12 November 2015) with SMI over 10 years, both community and in-patients, comprising mainly diagnoses of schizophrenia and bipolar illness, but also organic psychosis, psychotic depression and substance-induced psychosis. The data-set includes age, gender, ICD-10^21^ diagnosis, Community Health Index number, ethnicity, marital status, accommodation status and postcode, employment status, educational attainment, family history of psychosis, psychiatric admissions data, current illness severity (including Clinical Global Impression^22^ and Health of the Nation Outcome Scale scores^23^), current and previous medications and psychiatric comorbidities.

### Data linkage

**Fig. 1:**
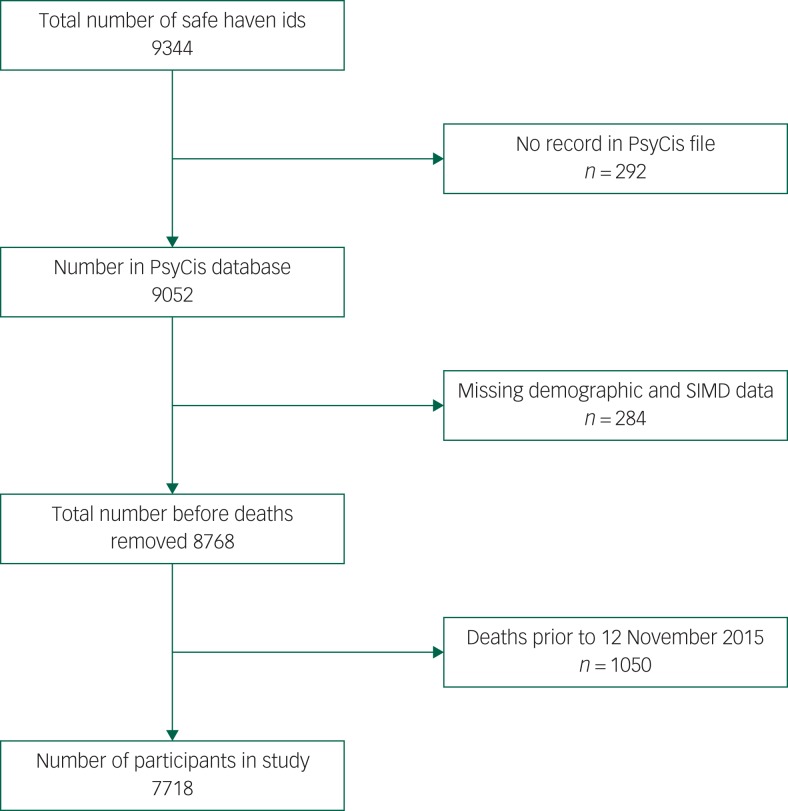
Flow chart of study numbers (Psychosis Clinical Information System, PsyCIS).

Data linkage to the Scottish Care Information store data repository enabled us to calculate by diagnosis, gender, age group and deprivation quintile the numbers of patients with no record, one, two and three recordings, for a range of blood markers. All data were linked over a 2-year period to the last recorded date in the PsyCis database (7718 patients on 12 November 2015). Those patients that had died during that period were removed from the data file prior to analysis (Fig. 1). We identified those who had at any time during that period had recorded an elevated glucose (≥11.1 mmol/L), elevated total cholesterol (≥5.0 mmol/L), low HDL cholesterol (≤1.0 mmol/L for men and ≤1.3 mmol/L for women) and an elevated HbA_1c_ (≥48 mmol/mol). We then linked these data to the encashed community prescriptions information system and calculated the numbers who were prescribed any drug under British National Formulary (BNF)^24^ section 3 (antidiabetic drugs) for raised glucose and raised HbA_1c_, or any lipid-regulating drug under BNF section 2.12 for raised total cholesterol and HDL cholesterol. Socioeconomic status was measured using the postcode-based Scottish Index of Multiple Deprivation (SIMD) by SIMD rank and divided into quintiles.

ids, identifications; SIMD, Scottish Index of Multiple Deprivation.

### Data analysis

We used Stata version 13.1 for statistical analysis. We used descriptive statistics to describe the population, such as mean values for continuous variables; counts (*n*), and proportions (%) were reported for categorical variables. Age- and gender-specific rates were generated for five age groups (16–34, 35–44, 45–54, 55–65, 65 and over). Odds ratios (ORs) from multivariable logistic regression were calculated with their 95% CIs to define any association between the OR of having a raised measure (dependent variable) and its associated factors (independent variables) gender, diagnosis, age groups and deprivation quintile, and also the OR of being on the appropriate medication if a raised measure had been recorded. A *P*<0.05 was considered for significant difference. We used complete case analysis for missing data for all demographic and SIMD analyses.

## Results

### Demographic and clinical characteristics

We identified data for a total of 7718 individuals on the PsyCIS database with complete sociodemographic and deprivation data (Fig. 1). Patients with schizophrenia comprised 40.9% of the cohort, bipolar disorder 24.2%, and ‘other psychotic disorders’ 34.9% (Table 1). A total of 56.1% of the cohort were male and 43.9% were female (Table 1). In total, 54% of patients in the PsyCIS cohort lived in the most deprived quintile of Glasgow compared with only 8.3% who lived in the most affluent quintile (see supplementary Table 1; available at https://doi.org/10.1192/bjo.2019.76).

**Table 1:** Routine blood monitoring in the preceding 2 years, by diagnosis and gender

	Total, *n* (%)	No record of blood monitoring, *n* (%)	One blood test, *n* (%)	Two blood test, *n* (%)	Three or more blood tests, *n* (%)	Group difference *P*
Glucose						
Male	4327 (56.1)	805 (18.6)	939 (21.7)	922 (21.3)	1661 (38.4)	<0.001
Female	3391 (43.9)	496 (14.6)	682 (20.1)	750 (22.1)	1463 (43.1)	
Other diagnosis	2696 (34.9)	537 (19.9)	592 (22.0)	587 (21.8)	980 (36.4)	<0.001
Bipolar	1868 (24.2)	229 (12.3)	366 (19.6)	396 (21.2)	877 (47.0)	
Schizophrenia	3154 (40.9)	535 (17.0)	663 (21.0)	689 (21.9)	1267 (40.2)	
Cholesterol						
Men	4327 (56.1)	995 (23.0)	1105 (25.5)	1050 (24.3)	1177 (27.2)	0.686
Women	3391 (43.9)	761 (22.4)	838 (24.7)	849 (25.0)	943 (27.8)	
Other diagnosis	2696 (34.9)	785 (29.1)	723 (26.8)	623 (23.1)	565 (21.0)	<0.001
Bipolar	1868 (24.2)	353 (18.9)	436 (23.3)	491 (26.3)	588 (31.5)	
Schizophrenia	3154 (40.9)	618 (19.6)	784 (24.9)	785 (24.9)	967 (30.7)	
Albumin						
Men	4327 (56.1)	760 (17.6)	588 (13.6)	638 (14.7)	2341 (54.1)	<0.001
Women	3391 (43.9)	449 (13.2)	380 (11.2)	499 (14.7)	2063 (60.8)	
Other diagnosis	2696 (34.9)	493 (18.3)	338 (12.5)	361 (13.4)	1504 (55.8)	<0.001
Bipolar	1868 (24.2)	197 (10.6)	208 (11.1)	270 (14.5)	1193 (63.9)	
Schizophrenia	3154 (40.9)	519 (16.5)	422 (13.4)	506 (16.0)	1707 (54.1)	
HbA_1c_						
Men	4327 (56.1)	2951 (68.2)	600 (13.9)	274 (6.3)	502 (11.6)	0.391
Women	3391 (43.9)	2367 (69.8)	454 (13.4)	213 (6.3)	357 (10.5)	
Other diagnosis	2696 (34.9)	2014 (74.7)	324 (12.0)	136 (5.0)	222 (8.2)	0.001
Bipolar	1868 (24.2)	1289 (69.0)	261 (14.0)	113 (6.1)	205 (11.0)	
Schizophrenia	3154 (40.9)	2015 (63.9)	469 (14.9)	238 (7.6)	432 (13.7)	
Triglycerides						
Men	4327 (56.1)	998 (23.1)	1107 (25.6)	1048 (24.2)	1174 (27.1)	0.778
Women	3391 (43.9)	763 (22.5)	846 (25.0)	841 (24.8)	941 (27.8)	
Other diagnosis	2696 (34.9)	785 (29.1)	727 (27.0)	624 (23.2)	560 (20.8)	<0.001
Bipolar	1868 (24.2)	355 (19.0)	438 (23.5)	485 (26.0)	590 (31.6)	
Schizophrenia	3154 (40.9)	621 (19.7)	788 (25.0)	780 (24.7)	965 (30.6)	
Creatinine						
Men	4327 (56.1)	728 (16.8)	752 (17.4)	710 (16.4)	2137 (49.4)	<0.001
Women	3391 (43.9)	409 (12.1)	497 (14.7)	512 (15.1)	1973 (58.2)	
Other diagnosis	2696 (34.9)	472 (17.5)	464 (17.2)	395 (14.7)	1365 (50.6)	<0.001
Bipolar	1868 (24.2)	182 (9.7)	214 (11.5)	241 (12.9)	1231 (65.9)	
Schizophrenia	3154 (40.9)	483 (15.3)	571 (18.1)	586 (18.6)	1514 (48.0)	
ALT						
Men	4327 (56.1)	766 (17.7)	822 (19.0)	762 (17.6)	1977 (45.7)	<0.001
Women	3391 (43.9)	458 (13.5)	560 (16.5)	592 (17.5)	1781 (52.5)	
Other diagnosis	2696 (34.9)	495 (18.4)	490 (18.2)	425 (15.8)	1286 (47.7)	<0.001
Bipolar	1868 (24.2)	208 (11.1)	290 (15.5)	319 (17.1)	1051 (56.3)	
Schizophrenia	3154 (40.9)	521 (16.5)	602 (19.1)	610 (19.3)	1421 (45.1)	

HbA_1c_, haemoglobin A_1c_; ALT, alanine aminotransferase.

### Blood monitoring

No routine blood monitoring had been conducted in 16.9% of patients within the preceding 2 years. Individuals with ‘other psychotic disorders’ (19.9%, *P*<0.001) received the least screening followed by patients with schizophrenia (17.0%) whereas those with bipolar disorder received the most screening with 12.3% (Table 1). Monitoring of routine blood markers was poorer in male patients (18.6%) compared with females (14.6%, *P*<0.001) (Table 1) and younger age groups (26.5%) were twice as likely not to have a clinical record of routine blood monitoring than older groups (11.6%, *P*<0.001). About half of those not being screened were in the age group 16–44 years (see supplementary Table 2). Screening for cholesterol and triglyceride levels was poor for all diagnostic categories with cholesterol screening being particularly poor in ‘other psychotic conditions’ (29.1%) followed by schizophrenia (19.6%) and bipolar disorders (18.9%, *P*<0.001). Triglyceride screening showed a similar pattern with least screening in ‘other psychotic conditions’ (29.1%) compared with schizophrenia (19.7%) and bipolar conditions (19.0%, *P*<0.001). Screening for HbA_1c_ were poorly recorded for all diagnostic groups with ‘other psychotic conditions’ being (74.7%), bipolar disorder (69.0%) and schizophrenia (63.9%, *P* = 0.001).

### Abnormal blood parameters

#### Glucose

In total 588 patients (7.6%) of the total 7718 cohort had a record of a raised glucose measure ≥11.1 mmol/L in the preceding 2 years of monitoring (Table 2). Raised glucose levels ≥11.1 mmol/L were higher in those with schizophrenia (8.8%, OR = 1.29, 95% CI 1.05–1.58, *P* = 0.013), compared with the ‘other psychotic disorders’ category (6.3%) and results for bipolar disorder (7.6%, OR = 1.11, 95% CI 0.88–1.41, *P* = 0.391). No difference was found by gender but the proportion of patients with a raised glucose increased markedly with age. Raised glucose levels ≥11.1 mmol/L were highest in the oldest age groups (age 65 and over, 10.7%, OR = 4.22, 95% CI 2.77–6.42, *P*<0.001) (Table 2) compared with the youngest group (age 16–34, 2.7%) with levels similar to the general population.^25^ Of the total 588 patients with a raised glucose, 382 (65.0%) were on medication for diabetes (see supplementary Table 3).

**Table 2:** Number of patients with raised blood parameters (by gender, diagnosis, age and deprivation quintile)

	Total, *n*	Raised glucose (≥11.1 mmol/L), *n* (%)	OR^a^ (95% CI)	OR *P*	Raised cholesterol (≥5.0 mmol/L), *n* (%)	OR^a^ (95% CI)	OR *P*	Reduced HDL cholesterol (≤1.0^b^ mmol/L), *n* (%)	OR^a^ (95% CI)	OR *P*	Raised HbA_1c_ (≥48.0 mmol/mol), *n* (%)	OR^a^ (95% CI)	OR *P*
Total	7718	588 (7.6)			3717 (48.2)			2821 (36.6)			754 (9.8)		
Gender													
Women	3391	253 (7.5)	Ref. (1.00)		1743 (51.4)	1.00		1407 (41.5)	1.00		320 (9.4)	1.00	
Men	4327	335 (7.7)	1.10 (0.93–1.32)	0.271	1974 (45.6)	0.83 (0.76–0.92)	<0.001	1414 (32.7)	0.69 (0.63–0.76)	<0.001	434 (10.0)	1.17 (1.00–1.37)	0.057
Diagnosis													
Other diagnoses	2696	170 (6.3)	1.00		1151 (42.7)	1.00		836 (31.0)	1.00		207 (7.7)	1.00	
Bipolar	1868	141 (7.6)	1.11 (0.88–1.41)	0.391	996 (53.3)	1.36 (1.20–1.54)	<0.001	757 (40.5)	1.38 (1.21–1.56)	<0.001	183 (9.8)	1.16 (0.94–1.44)	0.168
Schizophrenia	3154	277 (8.8)	1.29 (1.05–1.58)	0.013	1570 (49.8)	1.30 (1.17–1.45)	<0.001	1228 (38.9)	1.43 (1.28–1.60)	<0.001	364 (11.5)	1.40 (1.16–1.68)	<0.001
Age group													
16–34	1139	31 (2.7)	1.00		383 (33.6)	1.00		294 (25.8)	1.00		35 (3.1)	1.00	
35–44	1611	87 (5.4)	1.96 (1.29–2.98)	0.002	735 (45.6)	1.60 (1.37–1.88)	<0.001	531 (33.0)	1.33 (1.12–1.58)	0.001	91 (5.7)	1.80 (1.21–2.68)	0.004
45–54	2348	190 (8.1)	3.03 (2.05–4.46)	<0.001	1293 (55.1)	2.35 (2.03–2.73)	<0.001	938 (40.0)	1.76 (1.50–2.06)	<0.001	234 (10.0)	3.34 (2.32–4.81)	<0.001
55–64	1757	188 (10.7)	4.18 (2.83–6.18)	<0.001	929 (52.9)	2.08 (1.78–2.44)	<0.001	733 (41.7)	1.83 (1.55–2.16)	<0.001	258 (14.7)	5.30 (3.68–7.62)	<0.001
65 and over	863	92 (10.7)	4.22 (2.77–6.42)	<0.001	377 (43.7)	1.39 (1.15–1.67)	0.001	325 (37.7)	1.51 (1.25–1.84)	<0.001	136 (15.8)	5.82 (3.95–8.56)	<0.001
Deprivation quintile													
Least deprived	643	42 (6.5)	1.00		349 (54.3)	1.00		226 (35.2)	1.00		48 (7.5)	1.00	
2	634	33 (5.2)	0.77 (0.48–1.24)	0.287	341 (53.8)	0.97 (0.77–1.21)	0.777	228 (36.0)	1.03 (0.82–1.30)	0.796	52 (8.2)	1.09 (0.72–1.65)	0.672
3	901	73 (8.1)	1.23 (0.83–1.84)	0.298	463 (51.4)	0.85 (0.69–1.05)	0.128	310 (34.4)	0.95 (0.77–1.18)	0.649	90 (10.0)	1.36 (0.94–1.97)	0.106
4	1398	113 (8.1)	1.21 (0.84–1.76)	0.309	674 (48.2)	0.77 (0.64–0.93)	0.007	501 (35.8)	1.04 (0.85–1.27)	0.708	153 (10.9)	1.47 (1.04–2.07)	0.028
Most deprived	4142	327 (7.9)	1.17 (0.83–1.64)	0.367	1890 (45.6)	0.69 (0.58–0.81)	<0.001	1556 (37.6)	1.13 (0.94–1.35)	0.187	411 (9.9)	1.30 (0.95–1.79)	0.104

a. Odds ratios adjusted for all other variables.

b. High-density lipoprotein (HDL) cholesterol ≤1.3 mmol/L for women.

#### Glycosylated haemoglobin

In total, 754 patients (9.8%) had a record of an elevated glycosylated haemoglobin (HbA_1c_), indicating underlying or poorly controlled diabetes (Table 2). Raised HbA_1c_ (≥48 mmol/mol) was greater in those patients with schizophrenia (11.5%, OR = 1.40, 95% CI 1.16–1.68, *P*<0.001) compared with ‘other psychotic disorders’ (7.7%), and for bipolar disorder (9.8%, OR = 1.16, 95% CI 0.94–1.44, *P* = 0.168) (Table 2). Levels of elevated HbA_1c_ increased markedly with age with the highest in those aged 65 and above (15.8%, OR = 5.82, 95% CI 3.95–8.56, *P*<0.001) and only for deprivation quintile 4 (10.9%, OR = 1.47, 95% CI 1.04–2.07, *P* = 0.028). Of the 754 with an elevated HbA_1c_, 572 (75.9%, 7.4% of total cohort) were on medication for diabetes (see supplementary Table 3).

#### Prevalence of diabetes mellitus

Overall for this sample of patients, the prevalence of diabetes identifiable by this methodology was approximately 8.0%. In total 588 patients (7.6% of total cohort) had an elevated glucose (≥11.1 mmol/L) and of these 382 (65.0%) were on medication for diabetes. An elevated glycosylated haemoglobin was found in 754 patients (9.8%) and of these 572 (75.9%) were on medication, levels of treatment similar to the general population.^25^

#### Total cholesterol/HDL cholesterol

Overall 3717 patients (48.2%) had a record of elevated cholesterol levels ≥5.0 mmol/L) (Table 2). An elevated cholesterol (≥5 mmol/L) was higher in those with bipolar disorder (53.3%, OR = 1.36, 95% CI 1.20–1.54, *P*<0.001), compared with ‘other psychotic disorders’ category (42.7%) and an elevated cholesterol was found in those with schizophrenia (49.8%, OR = 1.30, 95% CI 1.17–1.45, *P*<0.001), compared with ‘other psychotic disorders’ category (42.7%). Of the total number 23.9% were on lipid-regulating medication (see supplementary Table 3).

In total 2821 (36.6%) patients were found to have a low HDL cholesterol level (Table 2). More patients were found to have a low HDL cholesterol level with bipolar disorder (40.5%, OR = 1.38, 95% CI 1.21–1.56, *P*<0.001) compared with ‘other psychotic disorders’ category (31.0%), while 38.9% of patients with schizophrenia (OR = 1.43, 95% CI 1.28–1.60, *P*<0.001) were found to have a low HDL cholesterol level. Of these 1060 (37.6%) patients were on lipid-lowering medication.

## Discussion

This comprehensive data linkage study addresses a key knowledge gap in the monitoring and treatment of physical health problems in patients with SMI. The principal findings are that routine screening has improved in recent years, with only 16.9% of the cohort having no record of routine blood monitoring within the preceding 2 years. The most recent screening studies by Crawford *et al*^12^ (2011) found that only 59.8% of patients had a plasma glucose recorded and Barnes *et al*^11^ in 2006 found with only 28.0% of patients had received blood monitoring. However, overall monitoring was poorer for male patients, people with schizophrenia and for younger SMI patients (16–44) where 23.9% of individuals had no record of screening. In all age groups, blood monitoring for lipids and glycosylated haemoglobin was poor, especially for those aged 16–44.

In total 7.6% of the cohort had an elevated plasma glucose recorded within the last 2 years and 9.8% had a raised HbA_1c_ with both of these indices were notably raised in schizophrenia and older patients. Levels of diabetes identifiable by this methodology were approaching twice that for the general population.^25^ We found that lipid levels and use of lipid-lowering medication were generally high in our study. In total 48.2% of the cohort (bipolar disorder 53.3%) were found to have a record of a cholesterol level over the recommended level of 5.0 mmol/L, and 36.6% of the total (bipolar disorder 40.5%) with a HDL cholesterol ≤1.0 mmol/L. In the general population in Scotland, 51.9% of people were found to have a cholesterol level ≥5.0 mmol/L and 10.8% an HDL cholesterol ≤1.0 mmol/L.^26^ In our study 23.9% of people with a cholesterol ≥5.0 mmol/L were on lipid-lowering medication and 37.6% with a HDL cholesterol ≤1.0 mmol/L, compared with treatment levels of about 15% in the general population.^26^

### Strengths and limitations

The strength of our study is the use of novel health informatics to monitor the health outcomes of 7718 people with SMI. Electronic record linkage allowed the analysis of near real-time clinical and prescribing data alongside access to a large clinical population that can be difficult to reach by standard approaches. Recent developments allowing screening for diabetes using glycosylated haemoglobin^27^ proved important in helping to identify levels of morbidity in this population, although some problems exist with this method when used in specific populations.^27^ Limitations of this study include our inability to investigate the role of other associated risk factors for physical comorbidity such as weight problems, smoking, dietary intake or sedentary behaviour. We also recognise that a (very small) proportion of patients may have received blood sampling or treatment elsewhere. We did not have access to a suitable control group within the database. Therefore our findings were compared with results found in the general population rather than an equivalent comparison group particularly in NHS Glasgow and Clyde where additional significant health problems are recognised. Additionally, some patients with significant levels of mental illness may not have attended any health services and therefore would not have been screened by the PsyCIS system and included in our sample.

We estimated the prevalence of diabetes using a range of markers such as glycosylated haemoglobin levels and a single random blood glucose level rather than conventional investigations such as a standard fasting glucose level, 2 h post-prandial test, or two or more raised random plasma glucose levels (≥11.1 mmol/L).^28^ Although we looked at the last 2 years of records, we may have missed individuals who have been diagnosed with diabetes or hypercholesterolemia, received successful treatment and had plasma glucose or cholesterol levels below the threshold for detection by our method. Our figures for the prevalence of diabetes and hypercholesterolemia are therefore likely to be an underestimate of actual comorbidity. A recent systematic review found the estimated overall worldwide pooled prevalence of diabetes in people with SMI to be 11.3%.^29^ However studies in this systematic review, using only similar cross-sectional methods, found the pooled prevalence to be 9.2%, and within the geographical area of Europe to be 7.7%, compared with 8.0% in our study.

We found that people with bipolar disorder received better levels of care than those with schizophrenia. The reasons for this were not clear, although some patients with bipolar disorder may receive regular health monitoring for routine medication, such as therapeutic plasma levels of lithium therapy. These findings may also be specific to our study, or reflect the wide variation in clinical care found in patients with SMI.^29^

## Implications

We have demonstrated that a novel approach using health informatics and linkage of routine clinical data (in an urban clinical population in Scotland) has considerable potential to assist in the improvement of physical health outcomes for people with SMI. It can establish baseline practice at a system-wide level (in our case a complete health board delivering care to around 21% of the Scottish population) and help determine the type of quality improvement needed across the National Health Service (NHS) in order to deliver better identification, monitoring and treatment of comorbid physical illness for individuals with SMI. Our research is a first step towards using health informatics to establish exactly how well NHS services in their totality are performing against a set of predetermined clinical tasks, within a specified group of patients in need. If reproduced across the rest of the UK, this could form the basis against which the implementation of a complex intervention aimed at improving the physical health of people with SMI could be measured. Although we did not conduct a health economic analysis, the use of routine data in this way has the potential to be more cost-effective than large-scale clinical audits.

In summary, this comprehensive data linkage study supports the use of health informatics as a means for improving physical health outcomes in patients with SMI. It has highlighted a number of important implications for clinical practice, most notably that we will continue to miss screening for a substantial proportion of patients unless we make system-level changes. Future work should be more ambitious and should replicate this approach across the UK, for example, to identify regional variations in practice and local variations in numbers of patients with these needs, so that resources can be more efficiently targeted to help mitigate health inequalities.

## Supplementary material

For supplementary material accompanying this paper visit http://dx.doi.org/10.1192/bjo.2019.76. click here to view supplementary material
