# Supplementary material for: Health screening, cardiometabolic disease and adverse health outcomes in individuals with severe mental illness
Source: BJPsych Open. 2019 Nov 8;5(6):e97. doi: 10.1192/bjo.2019.76 (PMC6854356; doi:10.1192/bjo.2019.76)
Supplement: Supplementary file 1 [file S2056472419000760sup001.docx]

Supplementary Table 1: Routine blood monitoring by social deprivation.

|  | Total (%) | No record | One | Two | Three | Group Difference  p values |
| --- | --- | --- | --- | --- | --- | --- |
| **SIMD** | Number (%) | Number (%) | Number (%) | Number (%) | Number (%) |  |
| **Glucose** |  |  |  |  |  |  |
| Least deprived | 643 (8.3) | 99 (15.4) | 147 (22.9) | 149 (23.2) | 248 (38.6) | 0.015 |
| 2 | 634 (8.2) | 116 (18.3) | 125 (19.7) | 157 (24.8) | 236 (37.2) |  |
| 3 | 901 (11.7) | 165 (18.3) | 172 (19.1) | 189 (21.0) | 375 (41.6) |  |
| 4 | 1,398 (18.1) | 241 (17.2) | 332 (23.8) | 303 (21.7) | 522 (37.3) |  |
| Most deprived | 4,142 (53.7) | 680 (16.4) | 845 (20.4) | 874 (21.1) | 1,743 (42.1) |  |
|  |  |  |  |  |  |  |
| **Cholesterol** |  |  |  |  |  |  |
| Least deprived | 643 (8.3) | 142 (22.1) | 150 (23.3) | 170 (26.4) | 181 (28.2) | 0.009 |
| 2 | 634 (8.2) | 144 (22.7) | 146 (23.0) | 184 (29.0) | 160 (25.2) |  |
| 3 | 901 (11.7) | 206 (22.9) | 193 (21.4) | 250 (27.8) | 252 (28.0) |  |
| 4 | 1,398 (18.1) | 328 (23.5) | 379 (27.1) | 327 (23.4) | 364 (26.0) |  |
| Most deprived | 4,142 (53.7) | 936 (22.6) | 1,075 (26.0) | 968 (23.4) | 1,163 (28.1) |  |
|  |  |  |  |  |  |  |
| **Albumin** |  |  |  |  |  |  |
| Least deprived | 643 (8.3) | 111 (17.3) | 74 (11.5) | 85 (13.2) | 373 (58.0) | 0.179 |
| 2 | 634 (8.2) | 96 (15.1) | 84 (13.3) | 105 (16.6) | 349 (55.1) |  |
| 3 | 901 (11.7) | 133 (14.8) | 125 (13.9) | 138 (15.3) | 505 (56.1) |  |
| 4 | 1,398 (18.1) | 225 (16.1) | 196 (14.0) | 219 (15.7) | 758 (54.2) |  |
| Most deprived | 4,142 (53.7) | 644 (15.6) | 489 (11.8) | 590 (14.2) | 2,419 (58.4) |  |
|  |  |  |  |  |  |  |
| **HbA1c** |  |  |  |  |  |  |
| Least deprived | 643 (8.3) | 454 (70.6) | 81 (12.6) | 45 (7.0) | 63 (9.8) | 0.723 |
| 2 | 634 (8.2) | 447 (70.5) | 82 (12.9) | 46 (7.3) | 59 (9.3) |  |
| 3 | 901 (11.7) | 615 (68.3) | 126 (14.0) | 54 (6.0) | 106 (11.8) |  |
| 4 | 1,398 (18.1) | 973 (69.6) | 195 (14.0) | 77 (5.5) | 153 (10.9) |  |
| Most deprived | 4,142 (53.7) | 2,829 (68.3) | 570 (13.8) | 265 (6.4) | 478 (11.5) |  |
|  |  |  |  |  |  |  |
| **Triglycerides** |  |  |  |  |  |  |
| Least deprived | 643 (8.3) | 143 (22.2) | 149 (23.2) | 170 (26.4) | 181 (28.2) | 0.012 |
| 2 | 634 (8.2) | 144 (22.7) | 148 (23.3) | 182 (28.7) | 160 (25.2) |  |
| 3 | 901 (11.7) | 207 (23) | 194 (21.5) | 246 (27.3) | 254 (28.2) |  |
| 4 | 1,398 (18.1) | 328 (23.5) | 384 (27.5) | 322 (23.0) | 364 (26.0) |  |
| Most deprived | 4,142 (53.7) | 939 (22.7) | 1,078 (26.0) | 969 (23.4) | 1,156 (27.9) |  |
|  |  |  |  |  |  |  |
| **Creatinine** |  |  |  |  |  |  |
| Least deprived | 643 (8.3) | 91 (14.2) | 90 (14.0) | 111 (17.3) | 351 (54.6) | 0.437 |
| 2 | 634 (8.2) | 91 (14.4) | 107 (16.9) | 101 (15.9) | 335 (52.8) |  |
| 3 | 901 (11.7) | 133 (14.8) | 142 (15.8) | 148 (16.4) | 478 (53.1) |  |
| 4 | 1,398 (18.1) | 216 (15.5) | 250 (17.9) | 233 (16.7) | 699 (50.0) |  |
| Most deprived | 4,142 (53.7) | 606 (14.6) | 660 (15.9) | 629 (15.2) | 2,247 (54.3) |  |
|  |  |  |  |  |  |  |
| **ALT** |  |  |  |  |  |  |
| Least deprived | 643 (8.3) | 112 (17.4) | 109 (17.0) | 106 (16.5) | 316 (49.1) | 0.109 |
| 2 | 634 (8.2) | 101 (15.9) | 113 (17.8) | 125 (19.7) | 295 (46.5) |  |
| 3 | 901 (11.7) | 136 (15.1) | 170 (18.9) | 161 (17.9) | 434 (48.2) |  |
| 4 | 1,398 (18.1) | 229 (16.4) | 274 (19.6) | 267 (19.1) | 628 (44.9) |  |
| Most deprived | 4,142 (53.7) | 646 (15.6) | 716 (17.3) | 695 (16.8) | 2,085 (50.3) |  |

ALT=alanine aminotransferase

Supplementary Table 2 Routine blood monitoring by age group (all patients)

|  | **Total** | **No record of blood monitoring** | **One blood test** | **Two Blood test** | **Three or more blood tests** | **Group Difference**  **p values** |
| --- | --- | --- | --- | --- | --- | --- |
| Age group | Number (%) | Number (%) | Number (%) | Number (%) | Number (%) |  |
| **Glucose** |  |  |  |  |  |  |
| 16-34 | 1,139 (14.8) | 302 (26.5) | 284 (24.9) | 238 (20.9) | 315 (27.7) | <0.001 |
| 35-44 | 1,611 (20.9) | 356 (22.1) | 417 (25.9) | 297 (18.4) | 541 (33.6) |  |
| 45-54 | 2,348 (30.4) | 345 (14.7) | 496 (21.1) | 530 (22.6) | 977 (41.6) |  |
| 55-65 | 1,757 (22.8) | 198 (11.3) | 291 (16.6) | 424 (24.1) | 844 (48.0) |  |
| 65 and over | 863 (11.2) | 100 (11.6) | 133 (15.4) | 183 (21.2) | 447 (51.8) |  |
|  |  |  |  |  |  |  |
| **Cholesterol** |  |  |  |  |  |  |
| 16-34 | 1,139 (14.8) | 455 (40.0) | 330 (29.0) | 173 (15.2) | 181 (15.9) | <0.001 |
| 35-44 | 1,611 (20.9) | 495 (30.7) | 457 (28.4) | 310 (19.2) | 349 (21.7) |  |
| 45-54 | 2,348 (30.4) | 459 (19.6) | 571 (24.3) | 611 (26.0) | 707 (30.1) |  |
| 55-65 | 1,757 (22.8) | 230 (13.1) | 391 (22.3) | 556 (31.6) | 580 (33.0) |  |
| 65 and over | 863 (11.2) | 117 (13.6) | 194 (22.5) | 249 (28.9) | 303 (35.1) |  |
|  |  |  |  |  |  |  |
| **Albumin** |  |  |  |  |  |  |
| 16-34 | 1,139 (14.8) | 245 (21.5) | 152 (13.4) | 180 (15.8) | 562 (49.3) | <0.001 |
| 35-44 | 1,611 (20.9) | 339 (21.0) | 251 (15.6) | 245 (15.2) | 776 (48.2) |  |
| 45-54 | 2,348 (30.4) | 359 (15.3) | 294 (12.5) | 351 (15.0) | 1,344 (57.2) |  |
| 55-65 | 1,757 (22.8) | 185 (10.5) | 186 (10.6) | 247 (14.1) | 1,139 (64.8) |  |
| 65 and over | 863 (11.2) | 81 (9.4) | 85 (9.9) | 114 (13.2) | 583 (67.6) |  |
|  |  |  |  |  |  |  |
| **HbA1c** |  |  |  |  |  |  |
| 16-34 | 1,139 (14.8) | 913 (80.2) | 136 (11.9) | 38 (3.3) | 52 (4.6) | <0.001 |
| 35-44 | 1,611 (20.9) | 1,223 (75.9) | 190 (11.8) | 68 (4.2) | 130 (8.1) |  |
| 45-54 | 2,348 (30.4) | 1,578 (67.2) | 328 (14.0) | 171 (7.3) | 271 (11.5) |  |
| 55-65 | 1,757 (22.8) | 1,094 (62.3) | 263 (15.0) | 131 (7.5) | 269 (15.3) |  |
| 65 and over | 863 (11.2) | 510 (59.1) | 137 (15.9) | 79 (9.2) | 137 (15.9) |  |
|  |  |  |  |  |  |  |
| **Triglycerides** |  |  |  |  |  |  |
| 16-34 | 1,139 (14.8) | 457 (40.1) | 329 (28.9) | 174 (15.3) | 179 (15.7) | <0.001 |
| 35-44 | 1,611 (20.9) | 497 (30.9) | 461 (28.6) | 301 (18.7) | 352 (21.9) |  |
| 45-54 | 2,348 (30.4) | 460 (19.6) | 568 (24.2) | 618 (26.3) | 702 (29.9) |  |
| 55-65 | 1,757 (22.8) | 230 (13.1) | 397 (22.6) | 550 (31.3) | 580 (33.0) |  |
| 65 and over | 863 (11.2) | 117 (13.6) | 198 (22.9) | 246 (28.5) | 302 (35.0) |  |
|  |  |  |  |  |  |  |
| **Creatinine** |  |  |  |  |  |  |
| 16-34 | 1,139 (14.8) | 244 (21.4) | 239 (21.0) | 183 (16.1) | 473 (41.5) | <0.001 |
| 35-44 | 1,611 (20.9) | 323 (20.1) | 313 (19.4) | 272 (16.9) | 703 (43.6) |  |
| 45-54 | 2,348 (30.4) | 329 (14.0) | 387 (16.5) | 371 (15.8) | 1,261 (53.7) |  |
| 55-65 | 1,757 (22.8) | 177 (10.1) | 217 (12.4) | 285 (16.2) | 1,078 (61.4) |  |
| 65 and over | 863 (11.2) | 64 (7.4) | 93 (10.8) | 111 (12.9) | 595 (69.0) |  |
|  |  |  |  |  |  |  |
| **ALT** |  |  |  |  |  |  |
| 16-34 | 1,139 (14.8) | 246 (21.6) | 258 (22.7) | 192 (16.9) | 443 (38.9) | <0.001 |
| 35-44 | 1,611 (20.9) | 340 (21.1) | 350 (21.7) | 280 (17.4) | 641 (39.8) |  |
| 45-54 | 2,348 (30.4) | 367 (15.6) | 408 (17.4) | 418 (17.8) | 1,155 (49.2) |  |
| 55-65 | 1,757 (22.8) | 188 (10.7) | 248 (14.1) | 326 (18.6) | 995 (56.6) |  |
| 65 and over | 863 (11.2) | 83 (9.6) | 118 (13.7) | 138 (16.0) | 524 (60.7) |  |

ALT=alanine aminotransferase

Supplementary Table 3. Number (%) of patients with raised measurements on medication.

|  | **Raised Glucose on medication (≥11.1 mmol/L** | **Odds Ratios^1^**  **(95% CI)** | **Odds Ratio p value** | **Raised Cholesterol on medication (≥5.0 mmol/L** | **Odds Ratios^1^**  **(95% CI)** | **Odds Ratio p value** | **Reduced HDL Cholesterol on medication (<≤ 1.0^2^mmol/L)** | **Odds Ratios^1^**  **(95% CI)** | **Odds Ratio p value** | **Raised HbA1c on medication (≥48.0 mmol/mol)** | **Odds Ratios^1^**  **(95% CI)** | **Odds Ratio p value** |
| --- | --- | --- | --- | --- | --- | --- | --- | --- | --- | --- | --- | --- |
|  | Number (%) |  |  | Number (%) |  |  | Number (%) |  |  | Number (%) |  |  |
| Total | 382 (65.0) |  |  | 888 (23.9) |  |  | 1,060 (37.6) |  |  | 572 (75.9) |  |  |
| **Gender** |  |  |  |  |  |  |  |  |  |  |  |  |
| Women | 171 (67.6) | 1.00 |  | 412 (23.6) | 1.00 |  | 497 (35.3) | 1.00 |  | 248 (77.5) | 1.00 |  |
| Men | 211 (63.0) | 0.81 (0.57-1.16) | 0.253 | 476 (24.1) | 1.37 (1.16-1.62) | <0.001 | 563 (39.8) | 1.47 (1.24-1.75) | <0.001 | 324 (74.7) | 0.81 (0.57-1.15) | 0.238 |
| **Diagnosis** |  |  |  |  |  |  |  |  |  |  |  |  |
| Other | 102 (60.0) | 1.00 |  | 286 (24.9) | 1.00 |  | 303 (36.2) | 1.00 |  | 158 (76.3) |  |  |
| Bipolar | 91 (64.5) | 1.11 (0.69-1.78) | 0.674 | 232 (23.3) | 0.77 (0.62-0.95) | 0.016 | 264 (34.9) | 0.77 (0.61-0.97) | 0.024 | 135 (73.8) | 0.84 (0.52-1.34) | 0.454 |
| Schizophrenia | 189 (68.2) | 1.57 (1.04-2.36) | 0.032 | 370 (23.6) | 0.84 (0.69-1.01) | 0.062 | 493 (40.2) | 1.13 (0.92-1.38) | 0.232 | 279 (76.7) | 1.05 (0.70-1.58) | 0.822 |
| **Age group** |  |  |  |  |  |  |  |  |  |  |  |  |
| 16-34 | 19 (61.3) | 1.00 |  | 26 (6.8) | 1.00 |  | 26 (8.8) | 1.00 |  | 27 (77.1) | 1.00 |  |
| 35-44 | 47 (54.0) | 0.74 (0.31-1.72) | 0.480 | 98 (13.3) | 2.22 (1.41-3.49) | 0.001 | 99 (18.6) | 2.41 (1.52-3.82) | <.0.001 | 65 (71.4) | 0.79 (0.31-1.97) | 0.610 |
| 45-54 | 126 (66.3) | 1.32 (0.59-2.91) | 0.497 | 281 (21.7) | 4.15 (2.72-6.34) | <0.001 | 329 (35.1) | 5.88 (3.83-9.01) | <0.001 | 182 (77.8) | 1.14 (0.49-2.68) | 0.760 |
| 55-65 | 129 (68.6) | 1.43 (0.64-3.20) | 0.379 | 318 (34.2) | 8.34 (5.44-12.78) | <0.001 | 394 (53.8) | 13.78 (8.93-21.25) | <0.001 | 199 (77.1) | 1.08 (0.46-2.54) | 0.852 |
| 65 and Over | 61 (66.3) | 1.27 (0.54-3.01) | 0.579 | 165 (43.8) | 13.32 (8.44-21.03) | <0.001 | 212 (65.2) | 23.61 (14.75-37.81) | <0.001 | 99 (72.8) | 0.82 (0.34-1.99) | 0.665 |
| **Deprivation** |  |  |  |  |  |  |  |  |  |  |  |  |
| Least Deprived | 29 (69.1) | 1.00 |  | 68 (19.5) | 1.00 |  | 65 (28.8) | 1.00 |  | 37 (77.1) | 1.00 |  |
| 2 | 24 (72.7) | 1.30 (0.47-3.60) | 0.612 | 67 (19.7) | 1.03 (0.69-1.52) | 0.894 | 76 (33.3) | 1.25 (0.81-1.92) | 0.311 | 42 (80.8) | 1.34 (0.51-3.54) | 0.553 |
| 3 | 44 (60.3) | 0.66 (0.29-1.50) | 0.325 | 109 (23.5) | 1.38 (0.97-1.97) | 0.077 | 120 (38.7) | 1.55 (1.04-2.31) | 0.030 | 61 (67.8) | 0.62 (0.28-1.40) | 0.251 |
| 4 | 76 (67.3) | 0.93 (0.43-2.01) | 0.849 | 155 (23.0) | 1.28 (0.91-1.78) | 0.152 | 189 (37.7) | 1.47 (1.02-2.12) | 0.040 | 118 (77.1) | 1.02 (0.47-2.21) | 0.964 |
| Most Deprived | 209 (63.9) | 0.84 (0.42-1.70) | 0.633 | 489 (25.9) | 1.55 (1.15-2.10) | 0.004 | 610 (39.2) | 1.59 (1.14-2.22) | 0.006 | 314 (76.4) | 1.00 (0.49-2.04) | 0.991 |

^1^Odds ratios adjusted for all other variables.

^2^HDL Cholesterol ≤ 1.3 mmol/L for women.
